# Possible role of glial cells in the onset and progression of Lyme neuroborreliosis

**DOI:** 10.1186/1742-2094-6-23

**Published:** 2009-08-25

**Authors:** Geeta Ramesh, Juan T Borda, Amy Gill, Erin P Ribka, Lisa A Morici, Peter Mottram, Dale S Martin, Mary B Jacobs, Peter J Didier, Mario T Philipp

**Affiliations:** 1 Division of Bacteriology and Parasitology, Tulane National Primate Research Center, Covington, LA, USA; 2 Division of Comparative Pathology, Tulane National Primate Research Center, Covington, LA, USA; 3 Division of Veterinary Medicine, Tulane National Primate Research Center, Covington, LA, USA; 4 Department of Microbiology and Immunology, Tulane University Medical School, New Orleans, LA, USA

## Abstract

**Background:**

Lyme neuroborreliosis (LNB) may present as meningitis, cranial neuropathy, acute radiculoneuropathy or, rarely, as encephalomyelitis. We hypothesized that glia, upon exposure to *Borrelia burgdorferi*, the Lyme disease agent, produce inflammatory mediators that promote the acute cellular infiltration of early LNB. This inflammatory context could potentiate glial and neuronal apoptosis.

**Methods:**

We inoculated live *B. burgdorferi *into the cisterna magna of rhesus macaques and examined the inflammatory changes induced in the central nervous system (CNS), and dorsal root nerves and ganglia (DRG).

**Results:**

ELISA of the cerebrospinal fluid (CSF) showed elevated IL-6, IL-8, CCL2, and CXCL13 as early as one week post-inoculation, accompanied by primarily lymphocytic and monocytic pleocytosis. In contrast, onset of the acquired immune response, evidenced by anti-*B. burgdorferi *C6 serum antibodies, was first detectable after 3 weeks post-inoculation. CSF cell pellets and CNS tissues were culture-positive for *B. burgdorferi*. Histopathology revealed signs of acute LNB: severe multifocal leptomeningitis, radiculitis, and DRG inflammatory lesions. Immunofluorescence staining and confocal microscopy detected *B. burgdorferi *antigen in the CNS and DRG. IL-6 was observed in astrocytes and neurons in the spinal cord, and in neurons in the DRG of infected animals. CCL2 and CXCL13 were found in microglia as well as in endothelial cells, macrophages and T cells. Importantly, the DRG of infected animals showed significant satellite cell and neuronal apoptosis.

**Conclusion:**

Our results support the notion that innate responses of glia to *B. burgdorferi *initiate/mediate the inflammation seen in acute LNB, and show that neuronal apoptosis occurs in this context.

## Background

Lyme neuroborreliosis (LNB) is caused by the spirochete *Borrelia burgdorferi*. It manifests in 10–15% of untreated Lyme disease patients [1]. LNB affects both the peripheral and the central nervous systems (CNS), resulting in acute and chronic inflammation accompanied with neurological deficits that may persist for the lifetime of a patient [2]. Neuroborreliosis may present as meningitis, cranial neuropathy, transverse myelitis, acute radiculoneuropathy or, rarely, as encephalomyelitis [3]. Early symptoms after an acute attack of LNB may include severe headaches, chronic fatigue and flu-like symptoms, facial-nerve paralysis, and motor dysfunction presenting as acute ataxia with pain in the back and extremities of limbs, accompanied by cognitive disorders and depression [4].

A sign of acute meningitis of both bacterial and viral origin is migration of large numbers of leukocytes into the subarachnoid space, with such pleocytosis reaching values of 100 to 1000 cells per μL [5]. Under normal conditions, cerebrospinal fluid (CSF) contains 1–5 leukocytes per μL [6]. In meningitis caused by bacteria such as *Neisseria meningitidis, Haemophilus influenza*, or *Streptococcus pneumoniae *the local production of cytokines and chemokines by glial and endothelial cells upon contact with pathogens is currently regarded as the initial step in regulating the directed migration of distinct leukocyte populations to an inflammatory site within the CNS [7-10]. In Lyme meningitis the cellular sources of these mediators are unknown.

The CSF of LNB patients shows abnormalities within 3 to 6 weeks after infection, manifested as mononuclear pleocytosis, persistent plasma cells, intrathecal synthesis of *B. burgdorferi*-specific immunoglobulins and presence of *B. burgdorferi *DNA [11]. Immune mediators such as cytokines and chemokines implicated in playing a role in the pathogenesis of various inflammatory diseases of the nervous system have also been found in the CSF of LNB patients [12-19]. Further, microscopic evaluation of lesions from patients with LNB shows perivascular monocytic and lymphocytic cell infiltration concomitant with the presence of *B. burgdorferi *DNA [20,21].

Recently, we reported that the interaction of *B. burgdorferi *with rhesus monkey brain parenchyma elicits the inflammatory mediators IL-6, IL-8, IL-1beta and CXCL13 from glial cells, with concomitant oligodendrocyte and neuronal apoptosis [22]. In addition, primary cultures of microglia or astrocytes produced IL-6, TNF-alpha, IL-8, and the macrophage inflammatory proteins CCL3 and CCL4 in the presence of live *B. burgdorferi *[23]. Several of these mediators are associated with LNB [24,25], play a major role in the recruitment of leukocytes into the subarachnoid space in various types of infectious meningitis [26], and in the inflammatory response mounted by the CNS in other neurodegenerative diseases such as multiple sclerosis and experimental autoimmune encephalomyelitis [27,28]. We therefore reasoned that glial cells could be an early source of the cytokines and chemokines detected in the CSF during LNB. We further anticipated that this inflammatory context could potentiate glial and neuronal apoptosis, based on our earlier observations that documented that live *B. burgdorferi *induced inflammation and oligodendrocyte and neuronal apoptosis in brain explants *ex vivo*, and following intracerebral inoculation *in vivo *[22].

To address our hypotheses we devised an *in vivo *model of acute LNB in the rhesus monkey. Five rhesus macaques were given intrathecal inoculations with live *B. burgdorferi*, and 2 additional animals were given sham inoculations and served as controls. CSF and serum samples were serially collected over a 6-week period from 2 of the infected animals and one control, and over a 12-week period from the other 3 animals and the other control. Relative concentrations of cytokines and chemokines were determined in CSF and serum by multiplex and sandwich ELISA assays, to determine the immune mediators that were specifically associated with the initiation of Lyme meningitis along with the appearance of pleocytosis. CSF cell pellets, and tissues from various regions of the brain and spinal cord were cultured for spirochetes to evaluate the presence of active infection. Levels of anti-C6 antibodies [29] were determined in serial serum samples to monitor the appearance of the acquired immune response against *B. burgdorferi*. Histopathological evaluation, immunohistochemistry, immunofluorescence staining and confocal microscopy of sections from the brain, spinal cord, dorsal root ganglia (DRG) and heart tissues were performed post-necropsy to detect the presence of inflammatory lesions, *B. burgdorferi *antigen, and to identify the cells that produced immune mediators. We also looked for glial and neuronal apoptosis in the parenchyma of the brain and spinal cord, and in DRG by the *in situ *TUNEL assay. The results of these evaluations are described herein.

## Methods

### Spirochetal inoculum

*B. burgdorferi *strain B31 clone 5A19 spirochetes, passage one, isolated from an ear biopsy of a previously infected mouse, were grown in Barbour-Stoenner-Kelly (BSK)-H medium supplemented with 6% rabbit serum and antibiotics (Rifampicin at 45.4 mg/ml, Phosphomycin at 193 mg/ml and Amphotericin at 0.25 mg/ml) (Sigma, St. Louis, MO) to late logarithmic phase under microaerophilic conditions. Spirochetes were pelleted at 2000 × g for 30 min at RT. At the end of the run the rotor was left to coast without breaking so as to minimize damage to the live spirochetes. The culture was washed twice using phosphate buffered saline (PBS) pH 7.2 (Invitrogen, Grand Island, NY) and resuspended in RPMI 1640 medium (Biowhittaker, Walkersville, MD) to contain a suspension of 1 × 10^8 ^spirochetes/ml.

### Animals and intrathecal inoculation

Seven 3–7-year old male rhesus macaques (*Macaca mulatta*) of Chinese origin were used in this study. The inoculation protocol was approved by the Institutional Animal Care and Use Committee of the Tulane National Primate Research Center. Anesthetized animals received up to 1 ml of RPMI 1640 medium with 1 × 10^8 ^spirochetes (n = 5), or no spirochetes (n = 2) into the cisterna magna, after removal of an equivalent volume of CSF. This dose was found to be safe in studies involving intrathecal inoculation of *Treponema pallidum*, another spirochete, in rhesus macaques [30]. Similar inoculum doses were used previously in the rhesus monkey [31-34]. To monitor CNS inflammation following inoculation, CSF (0.5–1.0 ml) and serum (5 ml of blood) were collected on a weekly basis for 4 weeks, and then once every two weeks until the end of the study for each animal. Two inoculated animals (DR50, EL81) and one control (EL66) were necropsied at six weeks post-inoculation (PI) and three inoculated animals (DH50, EP51, CH82) and one control (EJ86) at 12 weeks PI. The procedure used for euthanasia was consistent with the recommendations of the American Veterinary Medical Association's Panel on Euthanasia.

### CSF cell enumeration, and *B. burgdorferi *culture

CSF samples were maintained on ice after collection until processed. Total cell numbers were determined using a Reichert Bright-Line metalized hemocytometer (Thermo Fisher Scientific, Waltham, MA) within 30 min of collection. For cytospin, preparations were made by centrifuging the CSF at 300 × g at 4°C for 10 min. The cell pellet was resuspended in 100 ml of fetal bovine serum, loaded in a Shandon EZ cytofunnel set up on positively charged slides, and spun at 700 rpm for 7 min with minimum acceleration on a Shandon Cytospin centrifuge (Thermo Fisher Scientific). Slides were air-dried and stained with Wright stain (Fisher Scientific, Pittsburg, PA), mounted, and stored at RT until evaluation. Slides were examined for cellular composition and cell morphology. A 100–200 nucleated-cell differential count was performed from multiple random areas of each slide under a 40 × objective. The CSF supernatant after removal of cells was collected and spun down at 8000 × g at 4°C for 20 min. The supernatant was aliquoted and frozen at -70°C for evaluation of immune mediators. The pellet was resuspended in 10 ml BSK-H medium, supplemented with 6% rabbit serum and antibiotics as described above (Sigma) for culture of *B. burgdorferi*.

### Detection of anti-VlsE (C6) antibodies in serum

Serum antibody levels to the VlsE C6 peptide (*B. burgdorferi *B31) were quantified by ELISA using a procedure described previously [29]. As is customary, the cut-off line for positive ELISA values was set at the mean value of all of the pre-immune serum specimens plus three times the standard deviation of that mean. The probability that an ELISA value above this cut-off line is negative is p = 0.003.

### Quantification of immune mediators in CSF and serum

CSF and serum cytokines and chemokines were quantified with the Bio-Plex Human Cytokine 17-Plex Panel following the manufacturer's instructions (Bio-Rad, Hercules, CA). The analytes detected by this panel are: Hu IL-1beta, Hu IL-2, Hu IL-4, Hu IL-5, Hu IL-6, Hu IL-7, Hu IL-8, Hu IL-10, Hu IL-12, Hu IL-13, Hu IL-17, Hu G-CSF, Hu GMCSF, Hu IFN-gamma, Hu MCP-1/CCL2, Hu MIP-1beta/CCL4, Hu TNF-alpha. The multiplex plate was read using a Bio-Plex 200 Suspension Array Luminex System (Bio-Rad). CXCL13 concentration was measured with a sandwich ELISA (R&D, Minneapolis, MN). As with the serology ELISA, the cut-off line for positive Bio-Plex and sandwich ELISA values was set at the mean value for each mediator of all of the pre-infection and control CSF specimens plus three times the standard deviation of that mean.

### Collection of tissues for histopathological evaluation, detection of intracytoplasmic localization of immune mediators, and detection of *B. burgdorferi *by culture

Tissues were collected from various regions of the brain and spinal cord and DRG, and fixed in formalin or Z-fixative (Anatech, Battle Creek, MI) for routine histopathological evaluation. For the purpose of detecting intracytoplasmic localization of cytokines, fresh unfixed tissues were collected from various regions of the brain and spinal cord, as well as DRG at necropsy, and immediately processed for blocking of intracytoplasmic cytokines (modification of the method previously described [35], by incubation of tissue slices for four hours in RPMI medium (Invitrogen) containing 10% fetal bovine serum (Invitrogen) and the fungal metabolite brefeldin A (Invitrogen), which blocks export of intracellular proteins via the endoplasmic reticulum. Tissues were then fixed in 2% paraformaldehyde in PBS pH 7.0 (USB, Cleveland OH), and cryopreserved as described previously [35]. Tissue samples from various regions of the brain and spinal cord were also collected at necropsy in BSK-H medium, supplemented with 6% rabbit serum and antibiotics (Sigma) for detection of *B. burgdorferi *by culture.

### Immunofluorescence staining of intracytoplasmic immune mediators, phenotypic markers of producer cells and of cells in inflammatory lesions

For *in situ *analysis, the cryopreserved tissues were cryosectioned into 16-μm sections as previously described [35]. Tissues collected in formalin or fixed with Z-fixative were also sectioned into 10–15 μm sections, depending on the availability of tissue. Presence of inflammatory lesions was determined by routine histopathological evaluation of tissues sectioned at 5 μm and stained with hematoxylin and eosin (H&E). Immunohistochemical staining was done using several monoclonal and polyclonal antibodies (anti-CD3, CD20, and CD68, Table 1). All histological sections were incubated with the primary antibody for 1 hour at room temperature, followed by biotinylated anti-rabbit or anti-mouse secondary antibodies (Dako) as appropriate for 30 minutes. Finally, sections were incubated with avidin-biotin complex (ABC) (Vector labs, Burlingame, CA) for 30 minutes, and the reaction was visualized with 3,3'-diaminobenzidine (Dako) as the chromogen. Sections were subjected to immunofluorescence staining as previously described [22]. The primary antibodies against various immune mediators, cell phenotypic markers and *B. burgdorferi *are listed in Table 1. Relevant isotype controls (Sigma) at the concentration of the corresponding primary antibodies were also included.

### Qualitative and quantitative assessment of glial and neuronal apoptosis

Glial and neuronal apoptosis was assessed by the *in situ-*TUNEL assay in tissues collected from the brain and spinal cord as well the DRG, that were directly fixed in 2% paraformaldehyde at collection and cryopreserved and frozen as previously described [22]. Sections were stained for any one of the following brain cell markers: NeuN (neurons), IBA-1 (microglia), GFAP-cy3 (astrocytes), or S-100 (astrocytes, oligodendrocytes, Schwann cells, satellite cells) by incubation with the appropriate primary antibody (Table 1) followed by secondary antibodies conjugated with Alexa Flour 568. Sections were fixed in 2% paraformaldehyde for 15 min, followed by a 15-min wash in PBS. Sections were then subjected to the terminal deoxynucleotidyl transferase mediated UTP nick end labeling (TUNEL)-ApopTagPlus fluorescein *in situ *apoptosis assay (Chemicon, Temecula CA) as per the manufacturer's instructions. Sections were also stained with anti-*B. burgdorferi *antibody as described above followed by secondary antibody conjugated to Alexa Fluor 633. Contiguous sections stained with isotype controls at the respective concentrations of the primary antibody were included. To confirm the identity of S-100-staining cells, S-100-stained (Alexa Fluor 568) sections were also stained with anti-GFAP followed by secondary antibody conjugated to Alexa Fluor 633 (blue), before doing the TUNEL assay. Cells that were only positive for S-100 (labeled red) were regarded as oligodendrocytes in the CNS and Schwann cells and/or satellite cells in the DRG [36], while those that were labeled pink due to an overlap of S-100 (red) and GFAP (blue) were considered to be astrocytes. Slides were washed and mounted as described above and stored at 4°C in the dark until viewed. The percentage of apoptotic cells from ten fields were counted from each section (more than 500 cells in all cases). The total number of NeuN or S-100 positive cells respectively in each section was ascertained, followed by the percentage of cells that colocalized with the TUNEL signal for each cell marker, All counts were made by viewing slides under a fixed magnification of 63 × (corresponding to an area of 0.05 mm^2^) using the confocal microscope (see below).

**Table 1:** Primary antibody and antibody/fluorochrome-conjugates against various immune mediators, *B. burgdorferi*, and cell phenotypes

**MEDIATOR/CELL TYPE**	**PRIMARY AB/SOURCE**	**ISOTYPE**	**DILUTION/CONCENTR.**
IL-6	anti-human IL-6 (Abcam Inc. Cambridge, MA)	Rabbit IgG	1:1000

IL-8	anti-human IL-8 RDI Flanders, NJ	Rabbit IgG	10 μg/ml

CCL2	anti-human CCL2 (BD)	Mouse IgG_1_	10 μg/ml
	
	anti-human CCL2 (Abcam)	Rabbit IgG	1:10

CXCL13	anti-human CXCL13 (R&D)	goat IgG	5 μg/ml

*B. burgdorferi*	anti-whole cell preparation (Accurate Chemicals, Westbury, NY)	Rabbit IgG	1:250
	
	anti- *B. burgdorferi*-FITC (Kirkegaard and Perry, Gaithersburg, MD)	Goat	1:10

Astrocyte	anti-human GFAP-cy3 (Sigma St Louis, MO)	Mouse IgG_1_	1:200
	
	anti-human GFAP (Sigma)	Mouse IgG_1_	1:200

Astrocyte, Oligodendrocyte, Schwann cell, satellite cell	anti-bovine S-100 (Sigma)	Rabbit IgG	1:1000

Microglia	anti-Iba1 synthetic peptide (Wako Pure Chemicals, Richmond, VA)	Rabbit IgG	1:100

Endothelial cells	anti-human GLUT-1 (Chemicon)	Rabbit IgG	1:50

Neuron	anti-mouse NeuN (Chemicon)	Mouse IgG_1_	1:20

T cell	anti-human CD3 (Dako, Carpinteria, CA)	Mouse IgG2a	1:10

B cell	anti-human CD20 (Dako)	Mouse IgG1	1:200

Macrophage	anti-human CD68 (Dako)	Mouse IgG1	1:50

### Confocal microscopy

Confocal microscopy was performed using a Leica TCS SP2 confocal microscope (Leica Microsystems, Exton, PA) as previously described [22]. Photoshop CS3 (Adobe systems Inc., San Jose, CA) was used for image processing.

### Statistical analysis

The statistical significance of the apoptosis data was evaluated using the one-way ANOVA non-parametric analysis, followed by the Tukey's stringency test using PRISM software (IBM).

## Results

### Intrathecal inoculation with *B. burgdorferi *results in active, persistent CNS infection

Live *B. burgdorferi *spirochetes were recovered from the CSF cell pellets of all but one of the inoculated animals at various time points PI, as follows: animals DR50 and EL81 at week 6, DH50 at week 2 and EP51 at week 10, except for animal CH82, whose CSF cell-pellet cultures yielded no spirochetes. Tissues harvested at necropsy from the dura mater and the cervical spinal cord of one of the two animals (EL81) that were inoculated with *B. burgdorferi *and euthanized 6 weeks PI were also culture positive for *B. burgdorferi*. CSF cell-pellet or tissue cultures of the control animals were negative throughout.

### Early appearance of pleocytosis and inflammatory mediators in CSF

**Figure 1:**
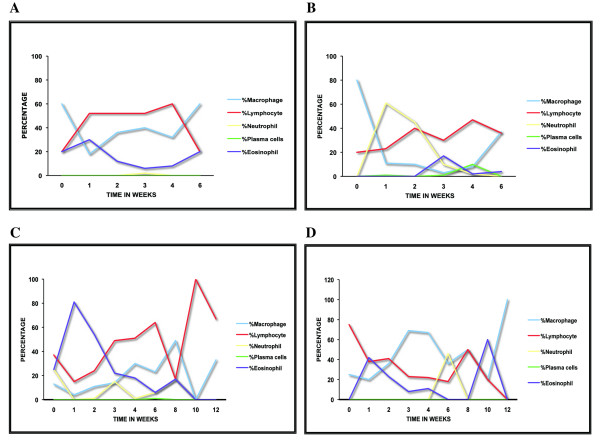
**CSF pleocytosis is primarily lymphocytic and monocytic**. Leukocyte differential count in CSF cytospin-smears showing the percentage of neutrophils, macrophages, lymphocytes, plasma cells and eosinophils found over time in animals with a pleocytosis higher than 50 cells/μL (A. EL81, B. DR50, C. DH50, D. EP51).

CSF pleocytosis was evident in all of the *B. burgdorferi-*inoculated animals as early as one week PI; specimens from the control animals were essentially free of cells (Table 2). Pleocytosis was minimal in CH82, which peaked to about 35 leukocytes/μl by week two PI. The evaluation of the percentage of various leukocytes (differential counts) of cytospins prepared from the CSF samples showed a predominance of lymphocytes and macrophage/monocytes. The differential counts of leukocytes in the CSF of all of the animals with a pleocytosis > 50 cells/μl is shown in Fig. 1.

**Table 2:** Cerebrospinal fluid pleocytosis

**ANIMAL**	**CEREBROSPINAL FLUID NUCLEATED CELL COUNT/μl**								
	
	**wk-0**	**wk-1**	**wk-2**	**wk-3**	**wk-4**	**wk-6**	**wk-8**	**wk-10**	**wk-12**
**DR50**	1	371	119	540	66	72			

**EL81**	0	133	62	22	19	9			

**EL66**	3	0	2	5	0	8			

**EP51**	0	32	84	25	15	12	5	0	3

**CH82**	0	8	35	11	7	2	1	0	0

**DH50**	5	213	114	0	92	161	40	10	1

**EJ86**	0	0	3	0	1	6	18	0	3

Total number of leukocytes/μl of CSF as a function of time PI, in animals DR50 and EL81 (up to 6 weeks PI) and EP51, CH82 and DH50 (up to 12 weeks PI) and respective controls (EL66, EJ86).

**Figure 2:**
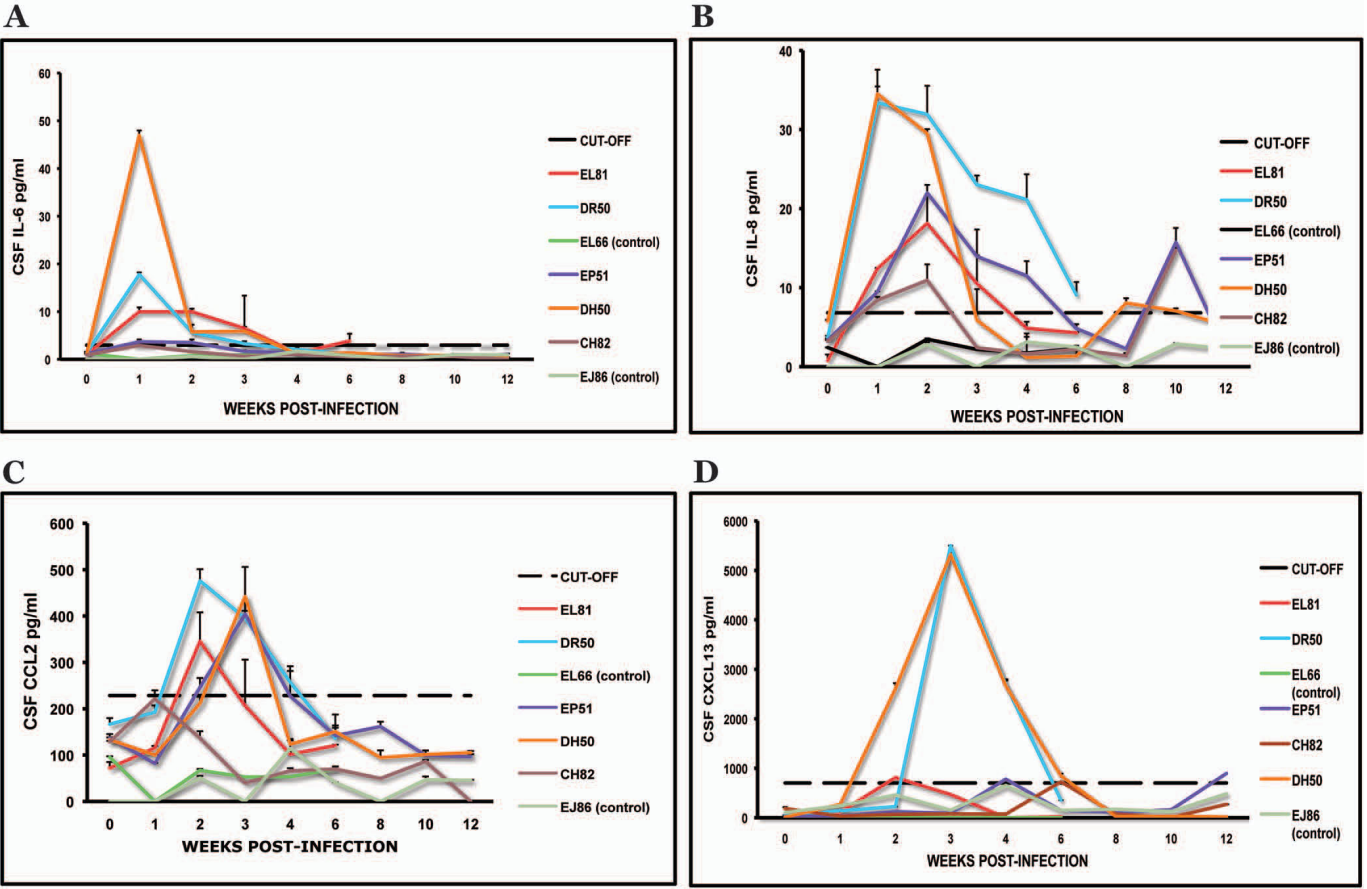
**Early appearance of cytokines and chemokines in the CSF**. Levels of IL-6 (A), IL-8 (B), CCL2 (C) and CXCL13 (D) in the CSF of all of the animals in the study as a function of time PI. The dotted line represents the cut-off value obtained as described in Materials and Methods. Data represent the mean ± SD of duplicate determinations except for CXCL13, where availability of CSF only permitted duplicate determinations for all data points of animal DH50, and some data points of animals DR50 and CH82.

CSF from infected animals showed levels above the cut-off value for the cytokine IL-6 in animals DR50, EL81 and DH50, between week 1–3 (Fig 2a). Animal EP51 showed levels of IL-6 just exceeding the cut-off value at weeks 1 and 2 PI. The chemokine IL-8 was significantly elevated in the CSF of all of the inoculated animals (Fig 2b), reaching maximum levels ranging from 10.9 to 35 pg/ml between weeks 1 and 2 PI. Monocyte chemoattractant protein-1MCP-1/CCL2 was significantly elevated in all of the infected animals (Fig 2c) except CH82, ranging between 350 pg/ml to 500 pg/ml by week 2 PI. The levels of IL-6 in the CSF were higher than those observed in the serum of all of the infected animals, except CH82, (Table 3), indicating that this cytokine was produced primarily intrathecally. On the other hand, IL-8 levels were much higher in serum than they were in CSF (Table 3). The levels of CCL2 were considerably higher in the CSF compared to those found at the corresponding time points in serum, suggesting, once again, a predominantly CNS origin for this chemokine. The concentration of the B-lymphocyte chemokine CXCL13/BLC was elevated above the cut-off value in animals DR50, DH50, EL81, and EP51, reaching peak values of around 5500 pg/ml between weeks 2 and 4 PI (Fig 2d). The CSF levels of CXCL13 were higher in three out of four of the inoculated animals (DR50, EL81, and DH50) as compared to those in paired serum specimens (Table 3), implying a possible CNS origin for this mediator as well. The very early appearance of inflammatory mediators (week 1–2 PI) in the CSF indicates that innate immune responses are at play. In addition, the very high serum concentrations of IL-8 detected as early as week 1 PI, much higher than the corresponding CSF levels, are data to suggest that the infection became systemic soon after the intrathecal inoculation.

**Table 3:** Peak CSF and corresponding serum values (pg/ml) of immune mediators in animals that were inoculated with *B. burgdorferi*.

**MEDIATOR**	**DR50**	**EL81**	**EP51**	**DH50**	**CH82**
**IL-6**	(wk-1)	(wk-1)	(wk-1)	(wk-1)	(wk-1)
**CSF**	17.8 ± 0.4	10 ± 0.9	3.7 ± 0.5	46.9 ± 0.2	2.9 ± 0.7
**SERUM**	0.7 ± 0.2	0.3 ± 0.05	1 ± 0.2	0	3.9 ± 0.5

**IL-8**	(wk-1)	(wk-2)	(wk-2)	(wk-1)	(wk-2)
**CSF**	33.4 ± 4.2	15.7 ± 0.1	21.9 ± 1.0	34.5 ± 0.9	10.9 ± 2.0
**SERUM**	832.8 ± 75	697.6 ± 86.2	625.6 ± 3.7	3442 ± 149.2	422.4 ± 25

**CCL2/MCP-1**	(wk-2)	(wk 2)	(wk-3)	(wk-3)	
**CSF**	475.9 ± 25.2	345.6 ± 62.3	404.5 ± 6.8	442.3 ± 6.8	*
**SERUM**	114.1 ± 6.5	71.8 ± 0.4	106.2 ± 2.8	39 ± 11.2	

**CXCL13/BLC**	(wk-3)	(wk-2)	(wk-4)	(wk-3)	
**CSF**	5500	811.5	779	5317.8 ± 70.5	*
**SERUM**	300	748	1221	199	

The time points at which peak values of various immune mediators (pg/ml) were observed in the CSF and corresponding values found in the serum of *B. burgdorferi*-infected animals are shown. Data represent the mean ± SD of duplicate determinations, except for CXCL13 in animals DR50, EL81, and EP51, where remaining CSF volume available only permitted a single determination.

* CSF concentrations of CCL2 and CXCL13 for animal CH82 were not significantly elevated with respect to control animals.

### Evaluation of the time of onset of the acquired immune response

**Figure 3:**
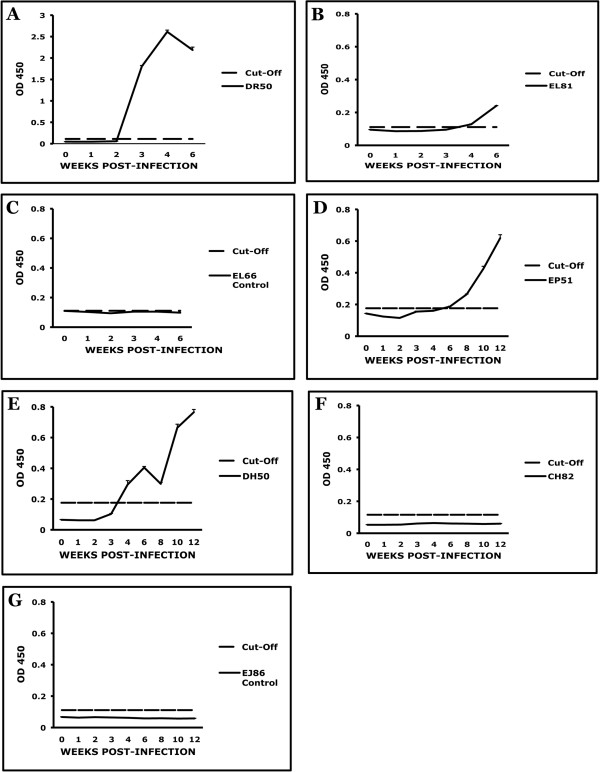
**Emergence of the acquired immune response only after week 3 PI**. C6 (VlsE) serum-antibody levels as a function of time PI, as measured by C6 ELISA in *B. burgdorferi*-infected animals. A. DR50, B. EL81, C. EL66, D. EP51, E. DH50, F. CH82, and G EJ86. The dotted line represents the cut-off value obtained as described in Materials and Methods. Data represents mean ± SD of triplicate determinations.

The evaluation of the time of onset of acquired immunity in the periphery was done by serially quantifying anti-VlsE serum antibodies. VlsE, the antigenic variation lipoprotein of *B. burgdorferi*, is expressed by the spirochete uninterruptedly upon the initiation of infection in mammals. We measured the anti-VlsE antibody response by quantifying antibodies to the VlsE C6 peptide. The earliest that this response was detected was by week 3 PI (animal DR50), Fig. 3A. In all of the other animals that had received a *B. burgdorferi *inoculation the anti-C6 response appeared between weeks 4 and 6 PI, except for animal CH82 (Fig. 3E), who did not have a detectable anti-C6 response. No anti-C6 response was detected in control animals (Fig. 3F, 3G).

### Histopathological evidence of Lyme meningitis, radiculitis, and inflammation in the DRG, along with evidence of *B. burgdorferi *antigen in both the CNS and peripheral nervous system (PNS) of infected animals

**Figure 4:**
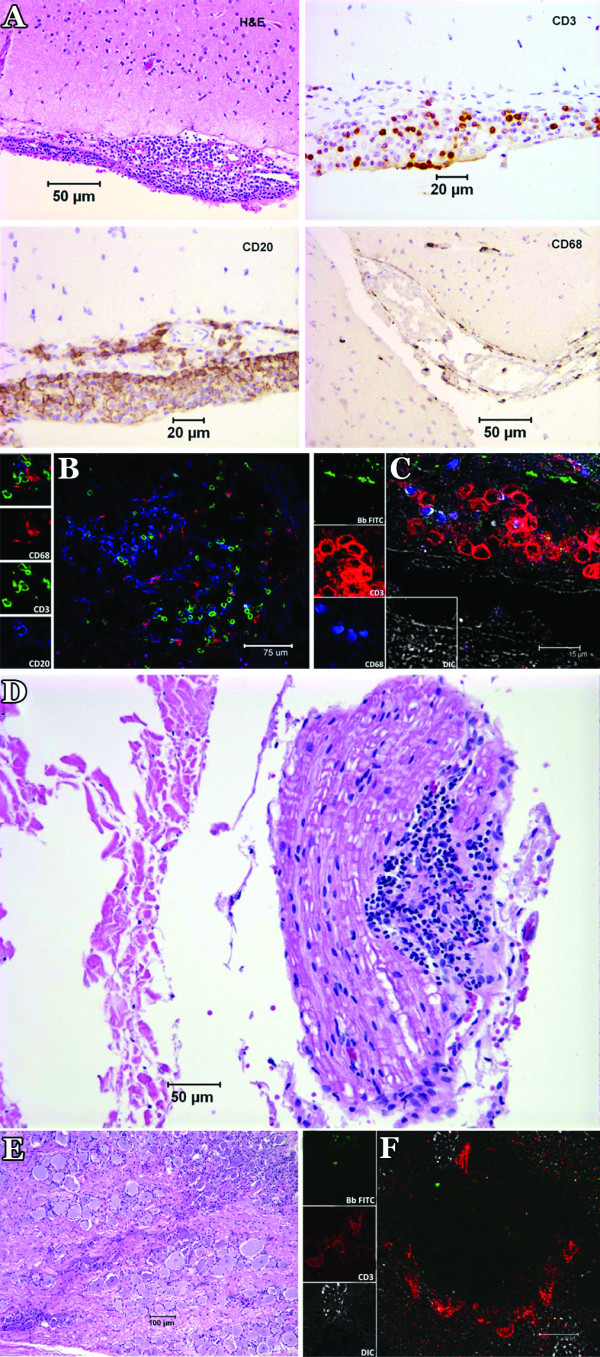
**Histopathological evaluation and immunofluorescence staining of lesions in the brain, dorsal root nerves and DRG**. A) Composite light microscope image of leptomeningitis in the brain of a *B. burgdorferi*-inoculated animal (DR50) showing lymphocytic, plasmacytic and monocytic infiltration by hematoxylin and eosin staining, as well as immunohistochemical staining showing CD3-labeled T cells, CD-20-labeled B cells, and CD68-labeled macrophages. B) Confocal micrograph showing immunofluorescence staining of CD3-labeled T cells (green), CD20-labeled B cells (blue) as well as CD68-labeling macrophages (red) in a representative inflammatory lesion from the meninges of animal DR50. C) FITC-labeled *B. burgdorferi *antigen (green) in the vicinity of CD3-staining T cells (red) and CD68-staining macrophages in lesions found in the frontal cortex of animal DR50. D) Radiculitis in the dorsal root of a cervical spinal nerve proximal to the ganglion of animal DH50. There is abundant lymphocytic, and monocytic infiltration as well as plasma cells. A few adjacent nerve fibers have swollen sheaths. E) Chronic-type inflammation of DRG from animal DH50 showing infiltrates of lymphocytes, plasma cells, and monocytes. F) *B. burgdorferi *antigen stained with antibody to *B. burgdorferi *labeled with FITC, in the vicinity of the CD3-staining T cells (red) in lesions found in DRG of animal DR50.

Histopathological evaluation of tissues collected from the 2 animals that were euthanized at 6 weeks PI revealed severe multifocal lymphocytic, monocytic and plasmacytic leptomeningitis in the brain and spinal cord. Radiculitis was evidenced by inflammatory infiltrates in the nerve roots of the cervical, thoracic, lumbar and sacral spinal cord. Similar lesions were seen in animal DH50, which was euthanized at 12 weeks PI. A representative image of brain leptomeningitis is shown in Fig 4A. The cellular infiltrates contained, in decreasing order of abundance, B lymphocytes (CD20^+^), T lymphocytes (CD3^+^), and monocyte/macrophages (CD68^+^). Figure 4B depicts a confocal micrograph of a lesion found in the dura mater of animal DR50 showing the presence of T cells, B cells and monocyte/macrophages. Inflammatory infiltrates in the vicinity of *B. burgdorferi *antigen were observed in the dura mater (not shown) and frontal cortex of animals DR50 (Fig. 4C), EL81 and DH50. As with the meninges, the parenchymal cell infiltrates were composed chiefly by B and T lymphocytes and few macrophages. *B. burgdorferi *antigen was also present in tissues collected from the periventricular areas and spinal cord of infected animals (not shown). Radiculitis seen as inflammation of the dorsal roots collected from the cervical, thoracic, lumbar, and sacral spinal cord was observed in animals DR50, EL81 (6-week-long infection) and DH50 (12-week). A representative image of radiculitis is depicted in Fig. 4D. Inflammation and presence of *B. burgdorferi *antigen was also observed in the DRG, in animals DR50 and DH50. A chronic type of inflammation of DRG from animal DH50 is shown in Fig 4E. The presence of *B. burgdorferi *antigen in the vicinity of CD3-labeling-T cells in the DRG is shown in Fig. 4F. No inflammatory lesions were detected at necropsy in the remaining animals that were given a *B. burgdorferi *inoculation and were euthanized at 12-weeks PI (EP51 and CH82), or in the control animals.

### Glial cells and neurons are among the producers of inflammatory mediators

**Figure 5:**
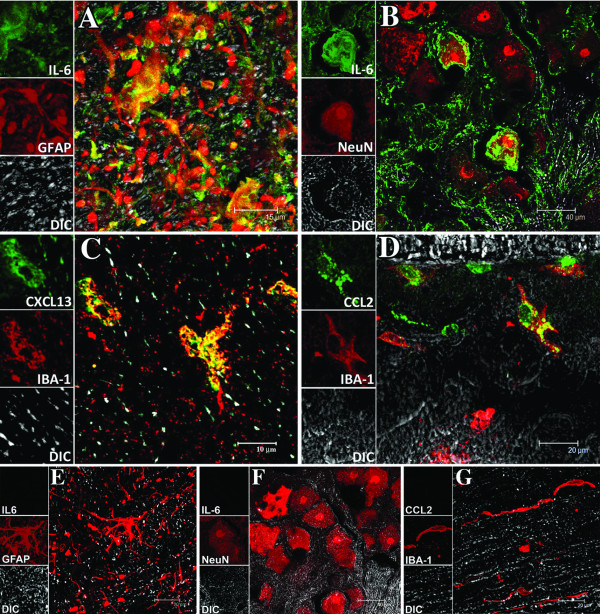
**Visualization of immune mediators and their producer cells in the CNS of *B. burgdorferi*-infected animals**. A) Indication of the cytokine IL-6 (green) due to staining with anti-IL-6 antibody followed by secondary antibody conjugated with Alexa fluor 488, in astrocytes (red), due to staining with anti-GFAP-cy3 in the spinal cord of animal DH50. The yellow signal is due to co-localization of the astrocytic marker GFAP (red) and IL-6 (green) within the astrocytes. B) The cellular localization of IL-6 (green) in neurons appearing red due to staining with anti-NeuN antibody, followed by secondary antibody conjugated with Alexa fluor 568. As NeuN is a nuclear antigen it is seen to stain the neuronal nucleus, while IL-6 is evident in the cytoplasm and extracellularly. C) The presence of CXCL13 (green) due to staining with anti-CXCL13 antibody followed by secondary antibody conjugated with Alexa fluor 488, in microglia (red) due to staining with anti-IBA-1 followed by secondary antibody conjugated to Alexa fluor 568 in the spinal cord of animal DH50. Intracytoplasmic staining of CXCL13 in microglia appears yellow. D) Evidence of CCL2 (green) due to staining with anti-CCL2 antibody followed by secondary antibody conjugated with Alexa fluor 488, in microglia (red) in the spinal cord of DH50. E). A representative image of the absence of IL-6 (which should have appeared green due to labeling with anti-IL-6 antibody followed by secondary antibody conjugated to Alexa fluor 488) in astrocytes labeled with anti-GFAP-cy3 (red) in the spinal cord of a control animal. F). Absence of IL-6 in neurons appearing red due to staining with anti-NeuN antibody, followed by secondary antibody conjugated with Alexa fluor 568 in the DRG of a control animal. G). Absence of CCL2 in microglia (red) due to staining with anti-IBA-1 followed by secondary antibody conjugated to Alexa fluor 568 in the spinal cord of control animals. The unstained tissue in all images appears gray due to differential interference contrast (DIC) imaging.

IL-6 was produced by both astrocytes and neurons, as visualized by intracytoplasmic staining of spinal cord tissues from three of the five *B. burgdorferi-*inoculated animals (DR50, EL81 and DH50). Fig. 5A shows IL-6 produced by astrocytes in the spinal cord. IL-6 was also found in neurons as well as extracellular in the DRG of animals DR50 and DH50 (Fig. 5B). CXCL13 and CCL2, two of the mediators produced chiefly in the intrathecal compartment, were localized to microglia in the spinal cord (Fig. 5C and Fig. 5D respectively). CXCL13 was also detected in CD68-labeled macrophages in the lesions (not shown). In addition, CCL2 and CXCL13 were present in endothelial cells, especially in the periventricular areas of the brain (not shown). IL-8 was not detectable in any of the tissues at the time of necropsy.

IL-6, CCL2 and CXCL13 were undetectable in comparable tissues from control animals (Fig. 5E, F, and 5G). No IL-6 was detected in astrocytes from the spinal cord or in neurons from the DRG of a control animal (Fig 5E and 5F, respectively). Similarly, microglia from the spinal cord of control animals were devoid of mediators (IL-6, IL-8, CCL2 or CXCL13). A representative image of microglia lacking CCL2 is shown in Fig 5G. Non-specific signals were absent in contiguous sections that were stained with immunoglobulin isotype controls at the concentrations of the respective primary antibodies, followed by the corresponding secondary antibodies.

### Glial and neuronal apoptosis

**Figure 6:**
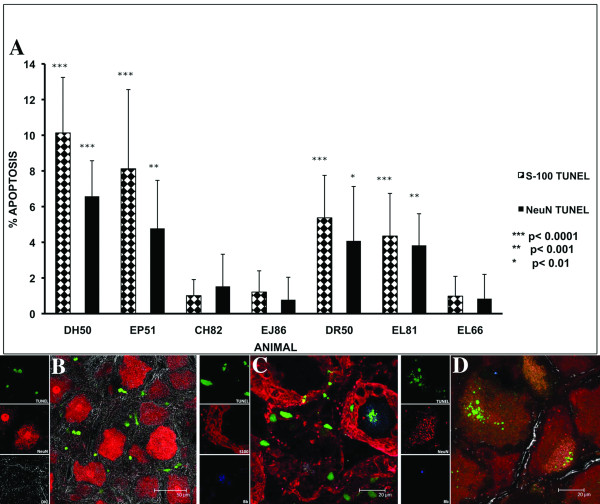
**Schwann cell and/or Satellite cell and neuronal apoptosis in DRG**. A) Percentage of Schwann/satellite cells and neurons dying by apoptosis as revealed by the *in situ *TUNEL assay performed on tissues from the DRG of all of the animals in the study. Data represent the mean values ± SD of the counts from ten fields. The p values represent the statistical significance with respect to the control animal (EL66 wk 6 and EJ86 wk 12) for each group. B) A qualitative view of the extent of the apoptosis (green) around neurons stained with antibody to NeuN followed by secondary antibody conjugated to Alexa fluor 568 (red) observed in the DRG of DH50 following the *in situ *TUNEL assay and immunofluorescence staining and confocal microscopy. C) Apoptosis of DRG Schwann/satellite cells. DRG sections from animal DH50 were labeled with antibody to S-100, followed by secondary antibody conjugated to Alexa fluor 568 (red). Cells undergoing apoptosis, stained using the TUNEL assay, are shown in green (FITC). Apoptotic Schwann/satellite cells surround a neuron that appears unstained as it does not take up the S-100 antibody, but is also showing the TUNEL signal in the center. D) Neurons stained with anti-NeuN antibody (red), and undergoing apoptosis (green) are shown in the vicinity of residual *B. burgdorferi *antigen (blue). The confocal micrograph is from a DRG section of animal DH50, following application of the *in situ *TUNEL assay and immunofluorescence staining. *B. burgdorferi *antigen is seen as blue due to labeling with anti-whole-cell *B. burgdorferi *antigen followed by secondary antibody conjugated to Alexa 633.

All of the infected animals except CH82 showed significant levels of both Schwann/satellite cell and neuronal apoptosis in the DRG as compared to that seen in the respective controls. The percentage of DRG Schwann/satellite cells (S-100^+^/GFAP^- ^staining cells) and neurons undergoing apoptosis in each of the animals in the study, as assessed by the *in situ *TUNEL assay is shown in Fig. 6A. Schwann/satellite cells showed significant levels of apoptosis ranging from 4.35 ± 2.39% (p < 0.0001) to 10.35 ± 3.11% (p < 0.0001). Neuronal apoptosis was less prevalent and ranged from 3.83 ± 1.77% (p < 0.001) to 6.58 ± 1.99% (p < 0.0001). Higher levels of both Schwann/satellite cell and neuronal apoptosis were observed in 2 of the animals necropsied at 12 weeks PI (EP51 and DH50) as compared to those necropsied at 6 weeks PI (Fig. 6A). A qualitative view of the extent of the apoptosis around neurons stained with antibody to NeuN observed in the DRG is shown in Fig 6B. Fig. 6C shows the TUNEL signal (green) seen in Schwann cells/satellite cells labeled with antibody to S-100 followed by secondary antibody conjugated to Alexa fluor 568 (red) that are surrounding a neuron that appears unstained as it does not take up S-100, but is also showing the TUNEL signal in the center and traces of *B. burgdorferi *antigen (blue) stained with antibody to whole *B. burgdorferi *followed by secondary antibody conjugated to Alexa fluor 633. Apoptotic DRG neurons in the vicinity of *B. burgdorferi *antigen are shown in Fig. 6D. No significant oligodendrocyte or neuronal apoptosis was detected in the tissues collected from the various regions of the brain and spinal cord. Apoptosis was not detected in astrocytes or microglia in any of the tissues.

## Discussion

This study depicts an *in vivo *model of acute LNB in the rhesus monkey. The model evidences the key features of CSF pleocytosis, as well as the classical lesions of leptomeningitis of brain and spinal cord and radiculitis that have been reported in patients with early LNB [2,4,11-15]. Importantly, live spirochetes were detected by culture of CSF cell pellets and CNS tissues collected post-mortem. This finding provides evidence of active CNS infection in this model.

Elevated CSF levels of both cytokines and chemokines have been reported in Lyme meningitis patients. The model not only reproduced these features but also provided, in addition, an insight into whether key inflammatory mediators did originate within the CNS or in the periphery, and an indication of the cellular sources of these mediators. That glia, and thus innate immune processes, are at least in part the source of relevant cytokines and chemokines was further suggested by the very early appearance of pleocytosis, likely caused by the enhanced concentration of these mediators in CSF within the first week PI. Acquired immunity, measured as serum antibody to VlsE, was only evident by week 3 PI, and later than week 3 in most animals. Perhaps the most significant finding was that of neuronal and Schwann/satellite cell apoptosis in DRG. As we discuss below, this phenomenon provides a probable etiology for Lyme neuroborreliosis of the PNS.

The evidence of pleocytosis by weeks 1–2 PI in all of the inoculated animals coincided with elevated levels of immune mediators (Table 1, Fig 2). This correlation indicates, but does not prove, that these immune mediators had a role in mobilizing the immune cells from the periphery into the CNS. The low levels of pleocytosis and immune mediators in animal CH82, as well as the lack of anti-C6 antibody response, could be due to an early clearance of active infection in this animal.

The ability to culture spirochetes from CSF cell pellets and CNS tissue and the occurrence of pleocytosis concomitant with an increase of immune mediators in the CSF implies that these phenomena occurred as a response to the presence of live spirochetes and spirochetal antigens in the CNS. The influx of immune cells from the periphery into the CNS is reflected in the cellular composition of the inflammatory lesions that were identified in the brain, spinal cord, dorsal root nerves and DRG of infected animals. The presence of *B. burgdorferi *antigen in CNS tissues and DRG located in the vicinity of inflammatory lesions indicates that the Lyme spirochete could have contributed to the induction of the acute inflammatory response both in the CNS as well as the PNS (DRG).

As chemokines are known to regulate inflammatory processes in the CNS in various bacterial and viral meningitides [26], acute Lyme meningitis could also be initiated via signaling mechanisms mediated by cytokines and chemokines, produced by glial cells in response to the Lyme spirochete. The presence of T cells and monocytes in lesions and in the CSF of spirochete-inoculated animals in this study, as well as those observed in the CSF of human patients with LNB, could be a consequence of the chemotactic action of the chemokine CCL2/MCP-1 [13,37,38]; this chemokine is also involved in enhancing production of inflammatory/immune cells from the bone marrow, an important feature that can assist in recruitment of these cells to the CNS. CCL2 was evident in the CNS as early as week 2 PI, and was shown to be produced by microglia. Moreover, its concentration in the CSF was up to 11 times higher than it was in the serum, in paired specimens of both fluids (Table 3); this is evidence to suggest that CCL2 was initially produced in the CSF. Considering that this chemokine has other important functions in the CNS, such as altering blood-brain barrier permeability [39], and affecting the survival of neuronal cells in other inflammatory neurodegenerative diseases such as multiple sclerosis and Alzheimer's disease [21,23,28,40-44], CCL2 could possibly be a contributor to the neurodegeneration [21,44] and vasculitis [3,44] observed in LNB.

The abundance of B cells in the lesions of spirochete-inoculated animals could be a consequence of the chemotactic activity of the B lymphocyte chemoattractant CXCL13. This chemokine was elevated in CSF by week 2 PI, and the fact that its concentration in CSF was in most cases higher than in paired serum specimens indicates, as with CCL2, that CXCL13 was primarily produced in the CNS. Our finding that microglia are among the cells that serve as a source of CXCL13 in the CNS corroborates our previous results that document the production of this chemokine by microglia in rhesus monkey brain explants that were exposed to *B. burgdorferi *[22]. It also echoes the observations of others who suggested a role for CXCL13 in LNB [24,25], and entails a possible driving force for the intrathecal B-cell infiltration and antibody production commonly observed in patients with spirochetal diseases.

The neutrophil attractant IL-8, which was detected in the CSF as early as week one PI, is likely a cause of the early accumulation of neutrophils in the CSF. Interestingly, even at this early time point, the concentration of IL-8 in serum was up to 100 times higher than that in the CSF (Table 3). No IL-8 was found in glial cells, in tissues collected at necropsy, even though this cytokine is produced *in vitro *both by astrocytes and microglia co-cultured with *B. burgdorferi *[23].

IL-6, which was already elevated in CSF by week one PI, could be contributed by glial cells, based on our observation of the localization of IL-6 to astrocytes, as well as to neurons. IL-6 has been documented to play a role in mediating inflammation in various inflammatory diseases of the nervous system [45-48] and in the pathogenesis of LNB [49,50]. Our observations affirm our previous report documenting astrocyte-derived IL-6 in response to live *B. burgdorferi *[22]. We have also previously shown that live *B. burgdorferi *as well as purified lipoproteins from this spirochete are able to induce the production of IL-6 in primary cultures of rhesus glial cells [23,51].

The role of microglia in initiating or promoting inflammatory processes in the CNS by facilitating the recruitment of peripheral immune cells has been well documented [52-54]. Here, we show that microglia can contribute the chemokines CCL2 and CXCL13 in the CNS and that the innate immune response mounted against the Lyme spirochete in the CNS, as evidenced by the appearance of immune mediators in the CSF, occurs before the onset of the acquired immune response. The latter was measured by the appearance of anti-C6 (VlsE) serum antibody. The induction of a syndrome indistinguishable from acute LNB prior to the onset of specific acquired immunity indicates that this syndrome can be induced by innate immune mechanisms, thus providing an insight into the pathogenesis of the acute form of the disease.

An important finding of this study is the significant Schwann/satellite cell and neuronal apoptosis in the DRG of *B. burgdorferi-*infected animals. This finding corroborates previous observations made in an *ex vivo *model of the interaction of live *B. burgdorferi *with brain explants from the frontal cortex of rhesus brain [22]. Our failure to find significant apoptosis in the brain and spinal cord in the *in vivo *model may be due to limited interaction of *B. burgdorferi *with parenchymal tissue, compounded with clearance of apoptotic cells by phagocytes such as macrophages and microglia [55]. In contrast, the detection of apoptotic cells in the DRG could be brought about by a delay in the recruitment of phagocytic cells to this area or by the presence of a larger concentration of neurons and Schwann/satellite cells per unit volume in the DRG as compared to neurons and oligodendrocytes in the parenchyma.

Mechanisms of neuronal damage as a consequence of bacterial meningitis have been studied extensively [56,57]. Neural injury has been documented in experimental neonatal meningitis due to group B streptococci [58]. The neurological sequelae of meningitis caused by *Streptococcus pneumoniae *also include neuronal dysfunction due to neuronal apoptosis [59]. Similar mechanisms of neuronal damage and neuronal dysfunction could be contributing to the cognitive and memory impairment seen in patients with LNB.

Neuroinflammation during bacterial meningitis is known to affect both neuronal synaptic plasticity as well as neuronal survival. Death of Schwann/satellite cells could also result in impaired neuronal function [60,61]. As we have shown that Lyme meningitis sets the stage for neuroinflammation and Schwann/satellite cell and neuronal damage, immune-mediated glial and neuronal damage could be among the underlying mechanisms of the cognitive and memory loss seen in LNB patients. The axonal damage observed in the PNS in LNB patients as well as in the rhesus monkey model of Lyme disease [62,63] could be a result of immune mediated death of DRG neurons, resulting in impaired nerve function.

Another potentially relevant finding of this study is the presence of IL-6 in neurons of DRG in infected animals. The induction of IL-6 in neurons of adult rat DRG that are adjacent to injured peripheral nerves has been shown to be a specific response to nerve injury [64,65]. IL-6 is suggested to play a critical role in affecting the activity of neurons in the DRG, increasing the receptiveness of neurons at nerve endings, axons as well as nerve cell bodies, resulting in lower back pain and sciatica [66]. IL-6-mediated signaling in glial cells in the spinal cord after spinal nerve injury is also known to play a role in the transduction of pain [67]. Thus the cytokine IL-6 produced in neuronal cells in the DRG as well as in glial and neuronal cells in the spinal cord that we observed in this model could be a possible factor contributing to the transduction of the lumbar pain that is often observed in patients with LNB.

## Conclusion

In conclusion, the findings of this study emphasize that glial cells are among the sources of a distinct set of immune mediators particularly the cytokines IL-6, and the chemokines CCL2 and CXCL13, in response to the Lyme spirochete in the CNS. Importantly, the observation of Schwann cell and/or satellite cell and neuronal apoptosis in the DRG as well as the inflammatory cytokine IL-6 in neurons in this region lend support to our hypothesis that the direct interaction of the spirochete with neural cells, a phenomenon that had long been predicted by experiments performed *in vitro *[68,69], and the ensuing immune response, may result in neurological damage. Our results are consistent with the notion that innate immune responses of glial cells to the Lyme disease spirochete initiate and/or mediate the inflammation that characterizes acute Lyme meningitis, and provide evidence of concomitant apoptotic cell death in the DRG. The latter could be the early event that leads to peripheral neuropathy in LNB.

## Competing interests

The authors declare that they have no competing interests.

## Authors' contributions

GR prepared the *B. burgdorferi *inoculum, processed the CSF samples for pleocytosis, differential white blood cell analysis and culture, processed necropsy samples, conducted sandwich ELISA assays for CXCL13, performed immunofluorescence staining and confocal microscopy, conducted the multiplex analysis of cytokines and chemokines from CSF and serum and drafted the manuscript. JTB and PJD performed the necropsies, processed samples for immunohistochemistry and conducted the routine histopathological evaluations of slides. AG conducted the evaluation of pleocytosis and differential counts. EPR was the veterinarian who oversaw the well being of the animals; she conducted the intrathecal inoculations and harvested CSF and serum samples. LAM conducted the software acquisition and analysis of the data for the multiplex assays. PM helped with immunohistochemistry and immunofluorescence staining and confocal microscopy. DSM conducted the culture of tissues at necropsy for *B. burgdorferi*. MBJ conducted the C-6 ELISA assays. MTP is the principal investigator for this project; he produced the basic project's design and directed and coordinated its realization. He also helped in drafting and preparing the manuscript for publication.
